# Natural Agents for the Improvement of Gingival Health: Systematic Review and Meta-Analysis of Randomized Clinical Trials

**DOI:** 10.3290/j.ohpd.c_2697

**Published:** 2026-06-19

**Authors:** Frederic Meyer, Zubaida Selva Mohammed, James Deschner, Joachim Enax

**Affiliations:** a Frederic Meyer Senior Scientist, Research Department, Dr. Kurt Wolff GmbH & Co. KG, Bielefeld, Germany. Conceived and designed the study, performed the study, data acquisition, data analysis, wrote the manuscript.; b Zubaida Selva Mohammed Dentist, International Academy RWTH Aachen University, Aachen, Germany. Conceived and designed the study, performed the study, reviewed and approved the manuscript prior to submission.; c James Deschner Professor and Chair, Department of Periodontology and Operative Dentistry, University Medical Center, Johannes Gutenberg University, Mainz, Germany. Conceived and designed the study, performed the study, reviewed and approved the manuscript prior to submission.; d Joachim Enax Senior Scientist, Research Department, Dr. Kurt Wolff GmbH & Co. KG, Bielefeld, Germany. Conceived and designed the study, performed data acquisition, reviewed and approved the manuscript prior to submission.

**Keywords:** clinical study, gingival health, natural agent, toothpaste.

## Abstract

**Purpose:**

This systematic review and meta-analysis evaluated the efficacy of natural ingredients used in toothpastes and gels in improving gingival health.

**Materials and Methods:**

A comprehensive search strategy using different databases was conducted in accordance with PRISMA guidelines to identify randomized clinical trials (RCTs) on the use of natural ingredients for improving gingival health. Included studies focussed on outcomes measuring gingival health parameters, such as gingival index (GI), or bleeding on probing (BoP). Out of 249 identified studies, 10 RCTs met the inclusion criteria. Formulations included natural ingredients from plant extracts such as *Aloe vera* or Pudilan, and hydroxyapatite (as tooth-like calcium phosphate).

**Results:**

The meta-analysis demonstrated a statistically significant improvement in gingival health when natural ingredients were used compared to control toothpastes and gels (p=0.0051). These findings suggest that natural ingredients may offer comparable or superior efficacy to conventional oral care products in terms of gingival health. Notably, *Aloe vera* and hydroxyapatite consistently demonstrated clinical benefits.

**Conclusions:**

Natural ingredients used in oral care products represent a promising strategy in gingivitis prevention and management. Their use in toothpastes and gels may therefore provide clinicians and patients with evidence-based, well-tolerated alternatives or adjuncts to conventional formulations for maintaining and improving gingival health.

Gingival health is clinically defined as an intact periodontium without inflammation or bone loss in non-periodontitis patients or patients with a history of periodontitis whose gingival status is currently stable.^⁠6^ This clinical framework has recently been complemented by biologically oriented concepts of periodontal diseases and host-response–based diagnostics, which may further refine disease classification and risk assessment.⁠^2,3,24^ Maintaining gingival health necessitates a comprehensive understanding of the disease etiology. Gingivitis is the precursor of periodontal disease (periodontitis), which is known to affect 20% to 50% of the global population.^43^ Long-term observational cohort data have further provided insight into the natural history and progression patterns of gingival inflammation to periodontitis in different populations.^30^ This disease is characterized by inflammation of the gingiva due to the accumulation of bacteria and debris between the gingival margin and the tooth; it is a reversible condition that can be improved by enhancing oral hygiene practices.⁠^7^ However, if left untreated, bacteria can penetrate deeper into the tissues and surrounding tooth structures, triggering a host response that, while defending against the invading bacteria, also destroys the periodontium, leading to periodontitis.^8,21^ Periodontitis is a chronic, destructive, and irreversible inflammatory disease state that, if not treated, can ultimately result in tooth loss.^20^

The World Workshop 2017 classified gingival diseases into two broad categories: dental plaque-induced gingivitis and non-dental plaque-induced gingival disease.⁠^6^ Beyond oral health, periodontal pathogens can also directly or indirectly promote the development of non-oral diseases. For instance, approximately 30 abundant species in the oral cavity, primarily Gram-negative anaerobic bacteria, are known to produce endotoxins that could directly contribute to systemic diseases.^44^ Periodontal disease has been linked to numerous systemic conditions, including diabetes mellitus, Alzheimer’s disease, rheumatic diseases, cardiovascular diseases, gastrointestinal diseases, respiratory infections, and adverse pregnancy outcomes.^49^

Evidence suggests a bidirectional association between diabetes mellitus and periodontal disease, in which diabetes increases the risk for periodontitis, and conversely, periodontal inflammation adversely affects glycaemic control.^28^ Additionally, periodontal treatment has been shown to improve diabetes symptoms, underscoring the importance of oral health for overall systemic health.⁠^4^ Periodontal disease is one of the two major dental diseases affecting human populations worldwide, with a high prevalence rate.^20,45^ Given its chronic nature, high prevalence, and impact on quality of life and systemic health, preventing and managing periodontitis has also been shown to be a value-for-money strategy from a health economic perspective, further emphasizing the importance of effective preventive approaches at the population level.^37^

While toothbrushing is known to remove dental plaque,^47^ the use of toothpaste with active ingredients (substances included to provide a specific, intended oral-health benefit beyond the basic function of the product) will be even more beneficial for gingival health.^17,32^ Dental plaque removal (dental plaque control) is important to prevent gingival and periodontal disease. Epidemiological data from recent national surveys highlight that despite increased awareness, periodontal disease remains highly prevalent and oral hygiene behavior and toothbrushing skills are still suboptimal in large parts of the population, underlining the need for effective and well-accepted preventive strategies.^13,15^ Besides dental plaque removal, inhibition of biofilm adhesion has also become an important option for prevention of gingival infections.^29^ In the past, chlorhexidine (CHX) has been used to treat and prevent gingival disease.^12^ Moreover, a variety of antiseptics and antibiotics have been evaluated as adjuncts to mechanical periodontal therapy, but their use is limited by concerns regarding side effects, ecological impact on the microbiota, and potential contribution to antimicrobial resistance.^19^ However, as concerns have been raised that CHX can lead to resistance-formation in bacteria against antibiotics, alternative treatments or prevention strategies are increasingly the focus of recent research.^10^ Those alternative agents are mainly natural ingredients including plant-derived or calcium-phosphate-based agents.^11,23,36^ Following this, the aim of the systematic review and meta-analysis was to identify natural ingredients used in oral care products, mainly toothpastes or gels, to prevent or treat gingival diseases.

## MATERIALS AND METHODS

### Study Design and Framework

The PICO framework was used to structure the focus of this literature review, ensuring a comprehensive and systematic approach to evaluating clinical studies where biomimetic agents were used in oral care products to improve gingival health. The PICO components were defined as follows:

P: Patients—The review included patients of all ages with mild to moderate periodontal conditions.
I: Intervention—The interventions considered were oral care products containing natural agents, i.e., plant-derived or tooth-like (hydroxyapatite).
C: Comparison—Comparisons were made between the groups (placebo or active control).
O: Outcome—The primary outcome measured was the improvement in gingival health, measured as bleeding on probing (BOP), gingival index (GI).

### Database Search and Selection Criteria

The primary database searched for relevant literature was PubMed (Ovid Medline). Additionally, Google Scholar was used to ensure a comprehensive search. The literature search adhered to the PRISMA guidelines for systematic reviews. The literature search covered publications up to and including May 10, 2025. Studies involving animals or in-vitro studies were excluded.

### Inclusion Criteria and Data Synthesis

We focused exclusively on human clinical trials and in-situ studies performed with humans that provided clinical evidence of efficacy in terms of improving gingival health or reduction of dental biofilms. Studies had to be double-blind randomized clinical trials with at least two treatment groups (natural active ingredients and placebo or active-control) and a duration minimum of 12 weeks. A qualitative synthesis was conducted for studies meeting the inclusion criteria.

Natural active ingredients were defined as those derived from vegetative plant parts (e.g., leaves), fruits, or minerals.

### Data Analysis 

All studies that met the inclusion criteria were reviewed and summarized (Table 1). Data that were extracted from the included studies were: authors and year of publication; natural agent(s); comparator; primary outcome/key results of the study; main conclusions.

**Table 1:** Overview of the study characteristics and results of the included studies

Author	Year	Title	Active ingredient(s)	Comparator	Results and conclusions
Figueiredo et al^18^	2024	The effect of a nature-based gel on gingival inflammation and the proteomic profile of crevicular fluid: a randomized clinical trial	Nature-based gel containing propolis, *Aloe vera*, green tea, cranberry, and *Calendula*	Conventional toothpaste	The test gel with the combination of different active ingredients improved the clinical status of gingivitis.
Vajrabhaya et al^46^	2024	Efficacy of a herbal toothpaste during active periodontal treatment: a clinical study	Herbal toothpaste containing *Aloe vera*	Abrasive toothpaste (active control) and conventional toothpaste (active control)	The herbal toothpaste with *Aloe vera* improved gingival health.
Butera et al^5^	2022	Home oral care of periodontal patients using antimicrobial gel with postbiotics, lactoferrin, and *Aloe barbadensis *leaf-juice powder vs conventional chlorhexidine gel: a split-mouth randomized clinical trial	Postbiotics gel with hydroxyapatite, lactoferrin, and *Aloe barbadensis *leaf-juice powder	Chlorhexidine gel	The biomimetic gel showed an improvement in gingival health (comparable to chlorhexidine).
Nandlal et al^35^	2021	A randomized clinical study to examine the oral hygiene efficacy of a novel herbal toothpaste with zinc over a 6-month period	Toothpaste with *Syzgium aromaticum, Aloe barbadensis, Emblica officinalis, Azadirachta indica, Ocimum baillicum,* and honey	Conventional toothpaste	The toothpaste with herbal ingredients and zinc was effective in improving gingival health.
Cheng et al^9^	2019	Evaluation of the effect of a toothpaste containing Pudilan extract on inhibiting plaques and reducing chronic gingivitis: A randomized, double-blinded, parallel controlled clinical trial	Toothpaste with Pudilan (contains several Chinese herbal ingredients) extract	Toothpaste without Pudilan	Pudilan-containing toothpaste is effective in reducing gingival inflammation and gingival bleeding.
Harks et al^22^	2016	Impact of the daily use of a microcrystal hydroxyapatite dentifrice on de novo plaque formation and clinical/microbiological parameters of periodontal health. A randomized trial	Toothpaste with hydroxyapatite	Antibacterial toothpaste (stannous fluoride and amine fluoride)	The toothpaste with biomimetic hydroxyapatite reduces gingival bleeding and improves overall gingival health.
Howshigan et al^26^	2015	The effects of an Ayurvedic medicinal toothpaste on clinical, microbiological and oral hygiene parameters in patients with chronic gingivitis: a double-blind, randomised, placebo-controlled, parallel allocation clinical trial	Toothpaste with different herbs: *Acacia chundra* (Willd), *Adhatoda vasica* (Nees.), *Mimusops elengi* L., *Piper nigrum* L., *Pongamia pinnate* (L.) Pierre, *Quercus infectoria* (Olivier.), *Syzygium aromaticum *L., *Terminalia chebula* (Retz.), *Zingiber officinale* (Roscoe)	Placebo toothpaste	The toothpaste with different herbs is effective in improving gingival parameters, such as bleeding on probing.
Hellström et al^25^	2014	The effect of a dentifrice containing *Magnolia *extract on established plaque and gingivitis in man: a six-month clinical study	Toothpaste with *Magnolia* extract (0.3%)	Placebo toothpaste	Toothpaste with *Magnolia* extract ameliorates gingivitis.
Namiranian et al^34^	2012	The effect of a toothpaste containing aloe vera on established gingivitis	Toothpaste with *Aloe vera*	Conventional toothpaste	Gingivitis was improved in both toothpaste groups.
Pradeep et al^41^	2012	Clinical and microbiologic effects of commercially available dentifrice containing aloe vera: a randomized controlled clinical trial	Toothpaste with *Aloe vera*	Triclosan-containing toothpaste (positive control), and placebo toothpaste (negative control)	*Aloe vera* toothpaste was effective in improving gingival health. The improvement was comparable to the triclosan group.


### Search Terms

**Fig 1:**
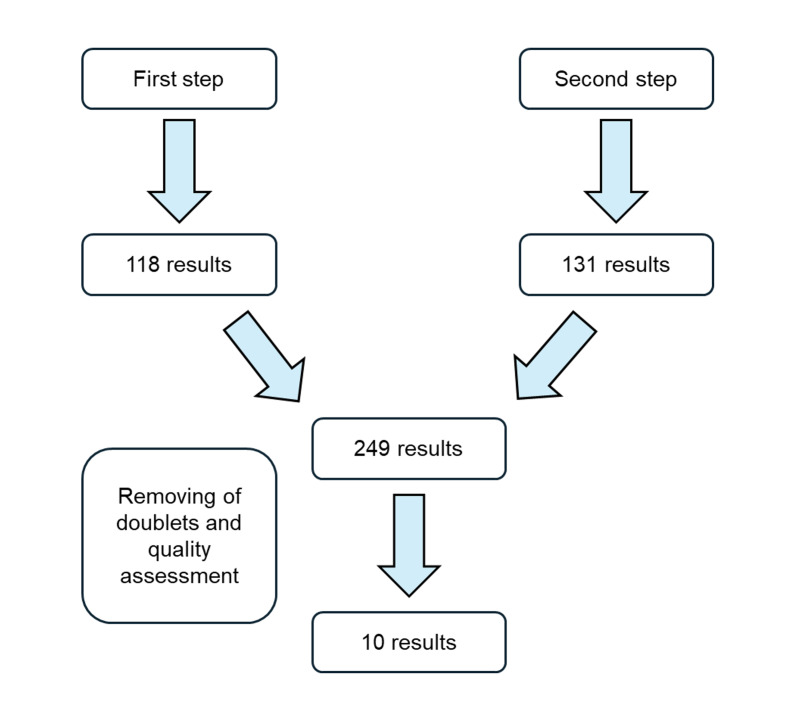
Schematic overview of the search results. The initial literature search (screening) resulted in 249 studies. After removing the doublets and quality assessment of the studies, according to the defined inclusion criteria, 10 studies remained for the further assessment.

Search terms and search strategy are given in Fig 1. A two-step strategy was employed.

First, we performed a broad search on all possible active ingredients that have been tested in clinical trials. The search terms were: (Clinical trial OR clinical study OR randomized trial) AND (gingivitis OR gingival disease OR gingival health OR periodontitis OR periodontal disease OR periodontal health) AND (biomimetic OR biomimetic AND (agent OR active OR ingredient) OR nature AND (identical OR extract) OR nature identical (ingredient OR active) OR plant OR plant extract) AND (oral care OR toothpaste OR toothbrushing OR tooth brushing OR dentifrice).

The second step focussed on searching active ingredients known from the literature (in-vitro or in-situ trials) or traditional treatments. The search terms were: (Clinical trial OR clinical study OR randomized trial) AND (gingivitis OR gingival disease OR gingival health OR periodontitis OR periodontal disease OR periodontal health) AND (Aloe Vera OR Curcumin OR Essential Oils OR hydroxyapatite OR Neem OR Propolis OR Salvadora persica OR Tea tree oil OR Triphala) AND (oral care OR toothpaste OR toothbrushing OR tooth brushing OR dentifrice).

### Meta-Analysis

#### Data extraction and data generation

Based on the data given in the studies, means and standard deviations at baseline and at the end of the study were used for the calculation. First, the difference between before and after (improvement or worsening) was calculated groupwise (active and control). Second, the difference between the groups was calculated. The calculated data were used for the meta-analysis.

Most studies used bleeding on probing (BoP) as a proxy for gingival health. In some studies, gingival index (GI) was applied but not BoP for the assessment of the gingival health. Consequently, BoP was used for data extraction and analysis, but when BoP was not reported, GI was used.

#### Statistical analysis 

All calculations and meta-analyses were performed using the open-source software R, version 4.5.1. In addition to the standard R packages, this study used the package “metafor” for the meta-analysis (random effects model) and forest plot.^48^

## RESULTS

After applying inclusion and exclusion criteria, 10 of 249 studies remained for further analysis. All 10 randomized clinical trials investigated the efficacy of natural oral care products (toothpastes or gels) in the improvement of gingival health (Table 1). The findings consistently demonstrate positive effects of natural ingredients on gingival health. However, compositions of the respective oral care products were not consistent, but rather multi-component formulations.

Figueiredo et al^18^ compared a gel containing propolis, *Aloe vera*, green tea, cranberry, and *Calendula*, to a conventional toothpaste as the active control (contains an established active ingredient). The test toothpaste statistically significantly improved clinical signs of gingivitis compared to the active control. Similarly, Vajrabhaya et al^46^ found that an *Aloe vera*-based herbal toothpaste improved gingival health during active periodontal treatment, outperforming both a standard control and a toothpaste with a higher amount of abrasives as active control toothpaste.

Butera et al^5^ demonstrated that an antimicrobial gel enriched with natural ingredients like postbiotics, lactoferrin, and *Aloe barbadensis* leaf juice, and hydroxyapatite was as effective as chlorhexidine (CHX) gel in improving gingival health, but with a more favourable side effect profile. Likewise, Nandlal et al^35^ reported improved gingival health with a herbal toothpaste containing *Syzygium aromaticum, Azadirachta indica*, and other medicinal herbs, supplemented with zinc, over a 6-month period.

Cheng et al^9^ also demonstrated statistically significant reductions in gingival inflammation and bleeding with the use of a Pudilan-based toothpaste (a combination of traditional Chinese herbal extracts, including *Scutellaria baicalensis Georgi, Isatis tinctoria* L., *Corydalis bungeana Turcz*, and *Taraxacum mongolicum Hand.-Mazz*) compared to a placebo. Harks et al^22^ found that a toothpaste containing hydroxyapatite statistically significantly reduced gingival bleeding and improved gingival health, with clinical benefits comparable to an antibacterial toothpaste containing stannous and amine fluorides.^22^ In another clinical trial, Howshigan et al^26^ observed improvements in clinical and microbiological parameters in patients with chronic gingivitis following the use of an ayurvedic herbal toothpaste. Furthermore, Hellström et al^25^ reported significant gingival improvement after six months of treatment with a toothpaste containing 0.3% magnolia extract. Namiranian et al^34^ showed clinical improvements in gingivitis for both *Aloe vera*-based and conventional home-use toothpaste groups. Pradeep et al^41^ confirmed the efficacy of an *Aloe vera*-based dentifrice in a randomized controlled trial, showing clinical improvements on gingival health comparable to triclosan-containing toothpaste.

**Fig 2:**
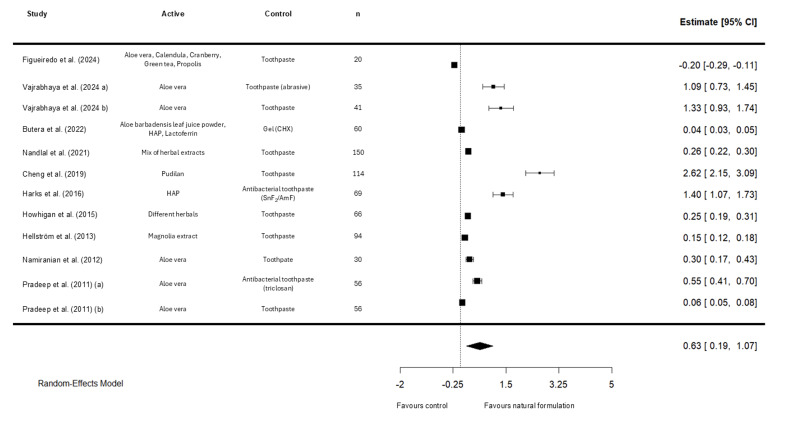
Meta-analysis of the studies with a natural ingredient compared to a positive control. The meta-analysis and random-effect model show statistically significant effects on gingival health for the natural ingredients contained in toothpastes or gels compared to the positive control group (p < 0.0001). Test toothpastes contained *Aloe vera*, hydroxyapatite, lactoferrin combined with hydroxyapatite and natural extracts or a mixture of different herbal extracts.

The meta-analysis confirms the positive effect of natural oral care products on gingival health. The random-effects model shows statistically significant results for the natural toothpastes and gels vs the control toothpastes and gels (p = 0.0051) (Fig 2).

## DISCUSSION

The results of recent clinical trials indicate that natural ingredients in toothpastes and oral gels may serve as effective alternatives in the improvement of gingival health or prevention of gingivitis. This has been proven by the meta-analysis using a random-effects model. Across all included studies, formulations containing natural bioactive ingredients such as *Aloe vera*, propolis, green tea, *Calendula*, *Magnolia* extract, Pudilan, hydroxyapatite, or a mixture of different herbal extracts were associated with statistically significant improvements in gingival health parameters.

The clinical benefit of *Aloe vera* was consistently demonstrated across several trials,^34,41,46^ where products containing this natural ingredient statistically significantly improved gingival health and, in some cases, performed comparably to well-established agents such as triclosan, CHX, or abrasive toothpastes.^5,41,46^ *Aloe vera* demonstrates several positive effects, including anti-inflammatory and wound-healing properties, attributed to compounds such as acemannan, but also antioxidant and antimicrobial activity.^42^

Multi-herbal combinations, such as the natural gel studied by Figueiredo et al^18^ and the ayurvedic toothpaste tested in the study by Howshigan et al,^26^ appeared particularly effective compared to the positive controls. These complex formulations may exert synergistic effects due to the complementary actions of multiple active phytochemicals.⁠^5^ However, the precise mechanisms of action remain to be fully elucidated and warrant further investigation, particularly via proteomic or microbiome-based analyses.

Interestingly, some natural products achieved an efficacy comparable to conventional active ingredients such as chlorhexidine⁠^5^ or stannous fluoride.^22^ Interestingly, both of those studies compared mineral hydroxyapatite, which is contained in oral care products, to well-established active ingredients, and in both studies, hydroxyapatite-containing products resulted in statistically significant improvement of gingival health.⁠^5,22^ This is particularly relevant given the known limitations of these conventional agents (chlorhexidine and stannous-compounds), such as taste disturbance, staining, or mucosal irritation with long-term use.^16,40^ Additionally, hydroxyapatite offers not only beneficial effects for gingival health, but has also been proven to prevent caries and remineralize the teeth as well as reduce hypersensitivity of teeth.^1,31,33,38^ The effect on improving gingival health might be due to its anti-adherent effects.^29,36^ Plant-based natural active ingredients such as *Aloe vera*, propolis extracts, or cranberry extracts are well known for their antibacterial and anti-infective properties.^14,27,39^ Products with natural ingredients may offer a better-tolerated and safer option for long-term management of gingival health, especially in sensitive populations (e.g., individuals with increased susceptibility to oral mucosal irritation or ingredient-related adverse effects, such as patients with pre-existing oral mucosal conditions). From a clinical perspective, such formulations may be particularly valuable as long-term maintenance options in patients with a history of gingivitis, those at increased inflammatory risk (e.g., due to orthodontic treatment or systemic conditions), or individuals who are intolerant of or reluctant to use conventional chemical actives.

Despite these promising findings, an important note on the study design should be considered. Firstly, heterogeneity in study design, duration, and outcome measures makes one-to-one comparisons challenging. Not all studies consistently used the same indices (GI, BoP), and follow-up durations ranged from 3 to 6 months. Secondly, few trials incorporated microbiological or immunological assessments that could clarify mechanistic pathways. However, this was not the focus of the studies but would help for a better understanding of the modes of action.

Future research is warranted, focusing on the inclusion of mechanistic endpoints—such as inflammatory biomarker expression, microbiota modulation, or salivary proteomics. By doing so, this would further strengthen the evidence base of the clinical outcome, but also the mechanistic understanding of the tested natural agents. Moreover, safety and tolerability profiles of herbal agents should be systematically assessed, particularly for formulations with multiple plant constituents. To test the efficacy of the natural ingredients in future studies, full qualitative and quantitative information on the composition should be given.

## CONCLUSION 

Natural-based toothpastes and gels represent a promising therapeutic strategy for managing gingivitis. The accumulated evidence supports their clinical efficacy, especially for patients seeking more natural alternatives to conventional oral care products. Clinically, these products may be considered as evidence-based alternatives or adjuncts within individualized preventive regimens, thereby broadening the range of available treatment options for dental professionals for the long-term maintenance and improvement of gingival health.

## ACKNOWLEDGMENTS AND CONFLICT OF INTEREST

FM and JE are employed as senior scientists in oral care at the Research Department, Dr. Kurt Wolff GmbH & Co. KG, Bielefeld, Germany. The other authors declare that they have no competing interests.

## References

[ref1] Amaechi BT, AbdulAzees PA, Alshareif DO, Shehata MA, de Carvalho Sampaio Lima PP, Abdollahi A (2019). Comparative efficacy of a hydroxyapatite and a fluoride toothpaste for prevention and remineralization of dental caries in children. BDJ Open.

[ref3] Buduneli N, Bıyıkoğlu B, Kinane DF (20002024). Utility of gingival crevicular fluid components for periodontal diagnosis. Periodontol.

[ref4] Bui FQ, Almeida-da-Silva CLC, Huynh B, Trinh A, Liu J, Woodward J (2019). Association between periodontal pathogens and systemic disease. Biomed J.

[ref8] Chapple ILC (2022). Time to take gum disease seriously. BDJ.

[ref9] Cheng L, Liu W, Zhang T, Xu T, Shu Y-X, Yuan B (2019). Evaluation of the effect of a toothpaste containing Pudilan extract on inhibiting plaques and reducing chronic gingivitis: A randomized, double-blinded, parallel controlled clinical trial. J Ethnopharmacol.

[ref10] Cieplik F, Jakubovics NS, Buchalla W, Maisch T, Hellwig E, Al-Ahmad A (2019). Resistance toward chlorhexidine in oral bacteria – Is there cause for concern. Front Microbiol.

[ref11] Cieplik F, Kara E, Muehler D, Enax J, Hiller K-A, Maisch T (2019). Antimicrobial efficacy of alternative compounds for use in oral care toward biofilms from caries-associated bacteria in vitro. Microbiol Open.

[ref12] Costo L, Mounsif M, Abdallaoui Maan L, Bouziane A (2025). Antiseptics prescription for the prevention and treatment of periodontal diseases: a comprehensive review. Health Sci Rep.

[ref13] Deinzer R, Jordan AR, Kuhr K, Margraf-Stiksrud J (2025). Oral hygiene behavior and toothbrushing skills: results of the 6th German Oral Health Study (DMS • 6). Quintessence Int.

[ref14] Duarte S, Rosalen PL, Hayacibara MF, Cury JA, Bowen WH, Marquis RE (2006). The influence of a novel propolis on mutans streptococci biofilms and caries development in rats. Arch Oral Biol.

[ref15] Eickholz P, Holtfreter B, Kuhr K, Dannewitz B, Jordan AR, Kocher T (2025). Prevalence of the periodontal status in Germany: results of the 6th German Oral Health Study (DMS • 6). Quintessence Int.

[ref16] Ellingsen JE, Eriksen HM, Rolla G (1982). Extrinsic dental stain caused by stannous fluoride. Scand J Dent Res.

[ref21] Hajishengallis G (2015). Periodontitis: from microbial immune subversion to systemic inflammation. Nat Rev Immunol.

[ref22] Harks I, Jockel-Schneider Y, Schlagenhauf U, May TW, Gravemeier M, Prior K (2016). Impact of the daily use of a microcrystal hydroxyapatite dentifrice on de novo plaque formation and clinical/microbiological parameters of periodontal health. A randomized trial. PloS one.

[ref24] Heitz-Mayfield LJA (2024). Conventional diagnostic criteria for periodontal diseases (plaque-induced gingivitis and periodontitis). Periodontol 2000.

[ref25] Hellström M-K, Ramberg P (2014). The effect of a dentifrice containing Magnolia extract on established plaque and gingivitis in man: a six-month clinical study. Int J Dent Hyg.

[ref26] Howshigan J, Perera K, Samita S, Rajapakse PS (2015). The effects of an Ayurvedic medicinal toothpaste on clinical, microbiological and oral hygiene parameters in patients with chronic gingivitis: a double-blind, randomised, placebo-controlled, parallel allocation clinical trial. Ceylon Med J.

[ref28] Jepsen S, Kebschull M, Deschner J (2011). Relationship between periodontitis and systemic diseases. Bundesgesundheitsblatt, Gesundheitsforschung, Gesundheitsschutz.

[ref29] Kensche A, Holder C, Basche S, Tahan N, Hannig C, Hannig M (2017). Efficacy of a mouthrinse based on hydroxyapatite to reduce initial bacterial colonisation in situ. Arch Oral Biol.

[ref31] Limeback H, Enax J, Meyer F (2023). Clinical evidence of biomimetic hydroxyapatite in oral care products for reducing dentin hypersensitivity: An updated systematic review and meta-Analysis. Biomimetics.

[ref32] Lippert F (2013). An introduction to toothpaste – its purpose, history and ingredients. Monogr Oral Sci.

[ref34] Namiranian H, Serino G (2012). The effect of a toothpaste containing aloe vera on established gingivitis. Swed Dent J.

[ref35] Nandlal B, Sreenivasan PK, Shashikumar P, Devishree G, Bettahalli Shivamallu A (2021). A randomized clinical study to examine the oral hygiene efficacy of a novel herbal toothpaste with zinc over a 6-month period. Int J Dent Hyg.

[ref36] Nobre CMG, König B, Pütz N, Hannig M (2021). Hydroxyapatite-based solution as adjunct treatment for biofilm management: An in situ study. Nanomater.

[ref37] Pattamatta M, Chapple I, Listl S (2025). The value-for money of preventing and managing periodontitis: Opportunities and challenges. Periodontol 2000.

[ref38] Pawinska M, Paszynska E, Amaechi BT, Meyer F, Enax J, Limeback H (2024). Clinical evidence of caries prevention by hydroxyapatite: An updated systematic review and meta-analysis. J Dent.

[ref39] Philip N, Walsh LJ (2019). Cranberry polyphenols: natural weapons against dental caries. Dent J (Basel).

[ref41] Pradeep AR, Agarwal E, Naik SB (2012). Clinical and microbiologic effects of commercially available dentifrice containing aloe vera: a randomized controlled clinical trial. J Periodontol.

[ref44] Socransky SS, Haffajee AD, Cugini MA, Smith C, Kent RL Jr (1998). Microbial complexes in subgingival plaque. J Clin Periodontol.

[ref45] Tonetti MS, Jepsen S, Jin L, Otomo-Corgel J (2017). Impact of the global burden of periodontal diseases on health, nutrition and wellbeing of mankind: A call for global action. J Clin Periodontol.

[ref47] Valkenburg C, Slot DE, Bakker EW, van der Weijden FA (2016). Does dentifrice use help to remove plaque? A systematic review. J Clin Periodontol.

[ref49] Winning L, Linden GJ (2015). Periodontitis and systemic disease. BDJ Team.

